# Cytochemical staining of leukocytes and platelets in the Florida manatee (*Trichechus manatus latirostris*): identification of a bilobed monocyte similar to other members of the *Paenungulata*

**DOI:** 10.3389/fvets.2023.1149000

**Published:** 2023-06-22

**Authors:** Laura A. Cagle, Nicole I. Stacy, John W. Harvey, Martine de Wit, Laurie Adler, Michael Walsh, Robert Bonde, Tracy Stokol

**Affiliations:** ^1^Department of Comparative, Diagnostic, and Population Medicine, College of Veterinary Medicine, University of Florida, Gainesville, FL, United States; ^2^Department of Physiological Sciences, College of Veterinary Medicine, University of Florida, Gainesville, FL, United States; ^3^Florida Fish and Wildlife Conservation Commission, The Marine Mammal Pathobiology Laboratory, St. Petersburg, FL, United States; ^4^Department of Large Animal Clinical Sciences, College of Veterinary Medicine, University of Florida, Gainesville, FL, United States; ^5^Department of Population Medicine and Diagnostic Sciences, Cornell University, College of Veterinary Medicine, Ithaca, NY, United States

**Keywords:** *Sirenia*, *Afrotheria*, cytochemistry, hematology, leukocyte morphology, white blood cells

## Abstract

Manatees (Antillean-, Amazonian, and African-) and dugongs belong to the Order *Sirenia*, and when combined with elephants and rock hyraxes, form the *Paenungulata*. A bilobed mononuclear cell has previously been identified in elephants and rock hyraxes, but not in manatees and dugongs, with cytochemical staining identifying these cells as bilobed monocytes in elephants. The objective of this study was to characterize leukocytes (white blood cells, WBC) and platelets in blood films of Florida manatees (*Trichechus manatus latirostris*; *n* = 8) using one routine hematological (Wright-Giemsa) and eight cytochemical stains: alkaline phosphatase (ALP), α-naphthyl butyrate esterase (ANBE), chloroacetate esterase (CAE), Luna, myeloperoxidase (MPx), periodic acid-Schiff (PAS), Sudan black B (SBB), and toluidine blue (TB). Heterophils and lymphocytes comprised most of the WBC, with low numbers of eosinophils, basophils, and monocytes. Additionally, 1–3% of the WBC were bilobed mononuclear cells. Bilobed mononuclear cell proportions were similar to rock hyraxes, but lower than elephants (approximate range 20–60%). Heterophils and eosinophils were positive for MPx, ALP, SBB, and PAS, with heterophils also being positive for CAE. Most of the lymphocytes were positive for ANBE and they were variably positive for CAE. Monocytes and bilobed mononuclear cells had similar cytochemical staining reactions (variably positive for all stains, except Luna and TB), supporting a monocytic origin, like elephants. Platelets were ANBE- and PAS-positive. Luna stain was useful for identifying eosinophils and TB was uninformative. This study provides new information on the morphological features and cytochemical staining characteristics of WBC and platelets and will aid in obtaining accurate hematological data of Florida manatees.

## Introduction

The Florida manatee (*Trichechus manatus latirostris*) is a subspecies of the West Indian manatee (*Trichechus manatus*) that inhabits Florida coastal waters. The species is listed as threatened by the US Fish and Wildlife Service under the Endangered Species Act (1, 2) and as endangered by the International Union for Conservation of Nature Red Book (3). Manatees, along with dugongs (*Dugong dugon*) belong to the Order Sirenia. Sirenians also belong to the clade *Afrotheria*, which is subdivided into *Afroinsectiphilia* (aardvarks, tenrecs, golden moles, elephant shrews) and *Paenungulata* (hyraxes, dugongs, manatees, elephants) (4, 5).

Baseline hematological evaluation of blood cell populations are essential in the general health assessment of any species and to monitor trends in individuals with disease. Blood film review is an integral part of the complete blood count (CBC) and relies on accurate blood cell identification. Hematological variables in elephants and manatees have been previously studied with reference data established (6–9). Harvey et al. (7) described the morphological features of mononuclear cells in the Florida manatee and identified small to intermediate lymphocytes with low numbers of granular lymphocytes, and monocytes with similar features to domestic mammals (in the current study referred to as “classic” monocytes). Eosinophils and heterophils can have similar staining properties with Romanowsky-type stains, so Medway et al. (10) applied a Luna stain to blood films of the West Indian Manatee to differentiate these two cell types. Medway et al. (10) confirmed that eosinophil granules are slightly larger, more uniform in size, and more refractile than those of heterophils. Manatee eosinophils cells also have a less lobulated nucleus and deeper blue staining cytoplasm than heterophils (10, 11).

A unique mononuclear cell type with a bi- or tri-lobed nucleus as well as “classic” monocytes have been identified in Asian (*Elephas maximus*) and African (*Loxodonta africana*) elephants and rock hyrax (*Procavia capensis*) within the *Afrotheria* clade (12, 13). Other species are suspected to have similar bilobed mononuclear cells, including the giant panda (*Ailuropoda melanoleuca*), flying fox (*Pteropus giganteus*), guinea pig, rabbit, and diamond python (*Morelia spilota*) (6, 12, 14–18).

As with elephants, a challenge with hematological evaluation of manatees relies on the proper differentiation of WBC during light microscopical evaluation. Cytochemical staining has been used to identify cellular constituents, such as lipids, carbohydrates, enzymes, and differentiate blood cell types in animals, when standard staining techniques (e.g., Wright-Giemsa, aqueous Romanowsky) are insufficient for cell classification (19, 20). The objective of this study was to characterize WBC and platelets in blood films of Florida manatees using standard and cytochemical staining techniques.

## Materials and methods

### Animals and blood sampling

This project was approved by the University of Florida’s Institutional Animal Care and Use Committee #202006823 and samples were collected under US Fish and Wildlife Service research permits #MA791721 and #MA067116-2.

Blood was collected from captured wild manatees from the brachial vascular bundle/arteriovenous plexus in the interosseous space between the radius and ulna into EDTA anticoagulant tubes [Becton Dickinson and Company (BD), Sparks, Maryland, United States] from eight clinically normal manatees during an ongoing health assessment study performed by the Florida Fish and Wildlife Conservation Commission and the US Geological Survey, Sirenia Project (21, 22). Blood samples were insulated and placed on ice packs immediately after collection until processing.

### Laboratory analysis and cytochemical staining

Blood films were prepared within 1 h after sampling at the field site. Blood was analyzed using an ADVIA 2120i (Siemens Healthcare Diagnostics, Tarrytown, NY) within 6–8 h after sample collection at the Diagnostic Laboratory of the University of Florida College of Veterinary Medicine within 24 h. Blood films were stained with Wright-Giemsa (Harleco ®, EMD Millipore, Billerica, MA, United States) and evaluated with light microscopy via performance of a 200-WBC differential count and morphological evaluation of WBC, erythrocytes, and platelets. WBC were classified as granulocytes (heterophils, eosinophils, basophils) and mononuclear cells (lymphocytes, “classic” monocytes, and bilobed mononuclear cells). Bilobed mononuclear cells were not differentiated from monocytes in a previous hematological study in manatees (7); however, they were differentiated from classic monocytes in this study because of their prominence in elephants. Reticulocytes and Heinz bodies were quantified as a percentage of total RBCs using a new methylene blue-stained blood film (Harleco ®, EMD Millipore, Billerica, MA, United States) (7).

Within 1 week after sampling, blood films were stained with eight cytochemical stains: alkaline phosphatase (ALP), α-naphthyl butyrate esterase (ANBE), naphthol AS-D choroacetate esterase (CAE), Luna, myeloperoxidase (MPx), periodic acid-Schiff (PAS), Sudan black blue (SBB), and toluidine blue (TB) at the Animal Health Diagnostic Center at Cornell University College of Veterinary Medicine. Commercially available kits (ALP, Procedure No. 85; ANBE, Procedure No. 181; CAE, Procedure No. 91; SBB, Procedure No. 380; Sigma-Aldrich, St. Louis, MO, United States) were utilized as previously described (14, 23). The myeloperoxidase assay was done manually by fixing the slides for 30 s in a buffer containing 0.35 mM disodium hydrogen phosphate, 1.4 mM potassium phosphate dibasic, 45% v/v acetone, and 10% formaldehyde (pH 6.6) then allowing them to air-dry. The slides were then incubated with the substrate for 30 min in the dark. The substrate consisted of 10 mg 3-amino-9-ethylcarbazole (Sigma-Aldrich) dissolved in dimethylsulfoxide in 50 mL of a buffer containing 3% v/v of 0.02 M glacial acetic acid and 7% v/v of 0.02 M sodium acetate. Hydrogen peroxide (0.006%) was added to the substrate buffer prior to incubation. The slides were counterstained with Gill’s hematoxylin for 30 s, then rinsed and air-dried. All slides were kept in the dark before and after staining. For the other stains, the slides were fixed with the kit fixative (citrate-acetone-formaldehyde for ANBE, CAE, and Sudan Black B or citrate-acetone for ALP). The Luna, PAS, and TB stains were performed via previously established protocols with histological sections of eosinophilic infiltrates, gastrointestinal tract, and a mast cell tumor as positive controls (14, 24, 25). Cytochemically stained blood films were evaluated by two independent reviewers (TS, NIS), using a 50 and 100x objective, to subjectively grade the staining pattern of each leukocyte type, similar to that described by Kehoe and colleagues for the giant panda (14). Pattern of staining was described as cytoplasm (C) or in granules (G), with degree of staining graded as positive (+), negative (−), equivocal (negative or weakly positive, +/−), weak (+), moderate (++), or strong (+++) positive. The pattern of cytoplasmic staining was further described as punctate (multiple small granular-like staining), focal (distinct foci), multifocal (multiple distinct foci), or diffuse (distributed throughout the cytoplasm). The numbers of each leukocyte observed during scanning varied depending on their frequency in blood, with few basophils being identified conclusively in the cytochemically-stained blood films. The results were then compared to obtain a consensus agreement on the frequency of observed cells by cell type in order to achieve evaluation of a sufficient number of cells. Images of the cells were taken with an Olympus BX53® (Tokyo, Japan) microscope equipped with a calibrated DP74® (Olympus, Tokyo, Japan) camera using the Olympus cellSens™ software (Standard Version 1.18, Tokyo, Japan).

## Results

**Figure 1:**
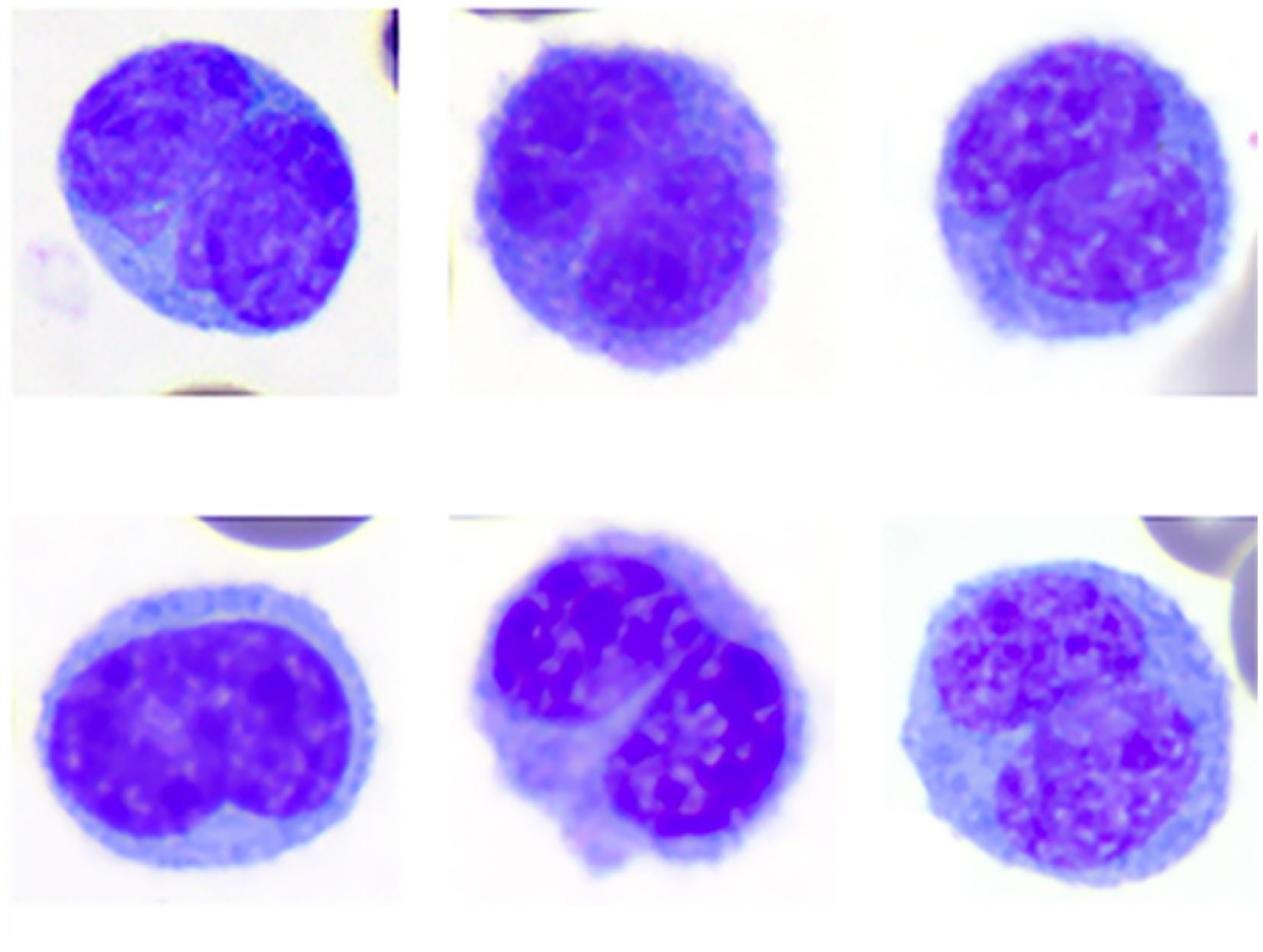
Image composite of monocytes of the Florida manatee (*Trichechus manatus latirostris*) showing the morphologic variability of bilobed monocytes. Wright-Giemsa-stain.

**Figure 2:**
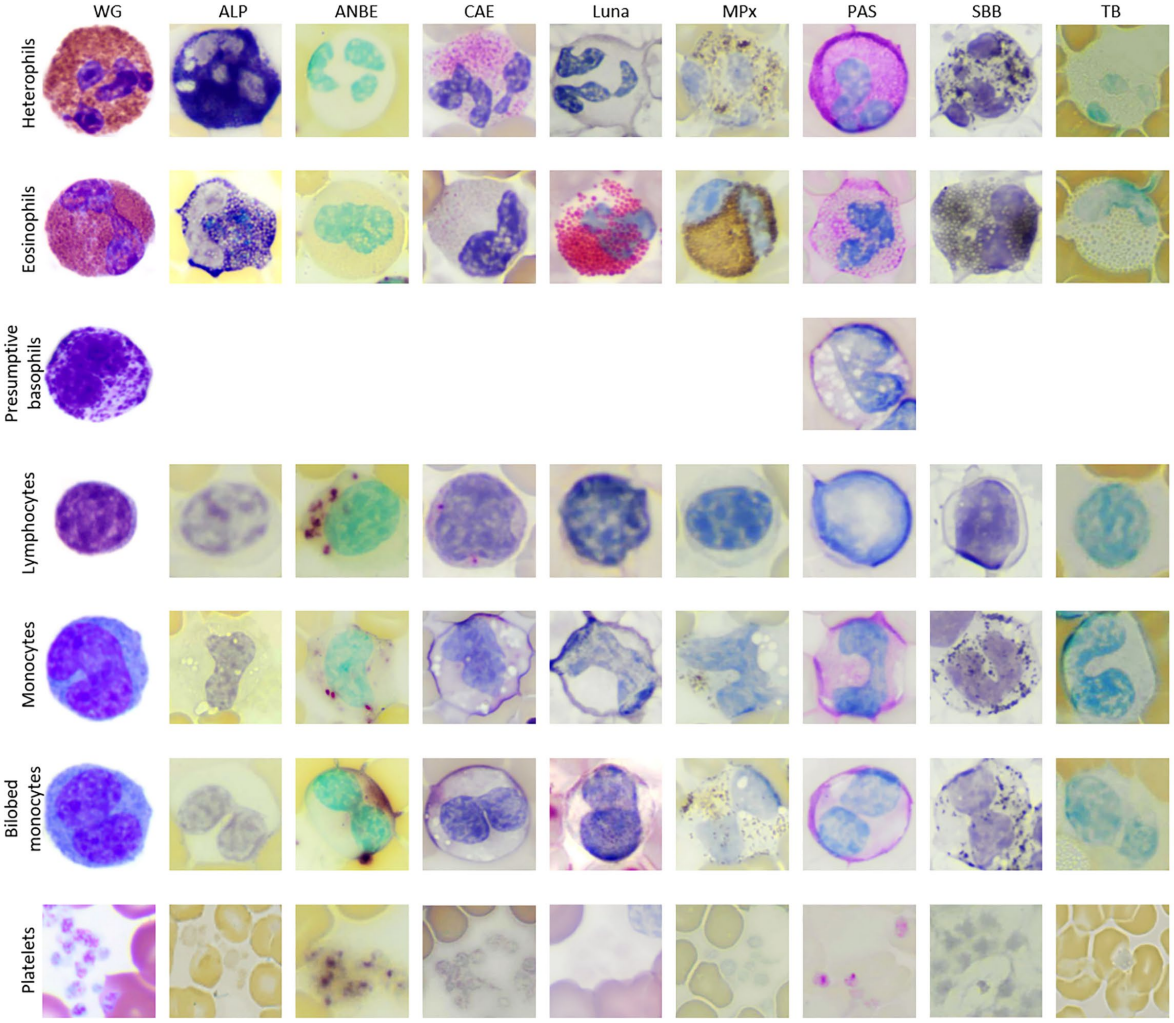
Image composite of leukocytes of the Florida manatee (*Trichechus manatus latirostris*) from Wright-Giemsa-stained blood films and blood films stained with eight cytochemical stains (ALP, alkaline phosphatase; ANBE, α-naphthyl butyrate esterase; CAE, chloroacetate esterase; Luna; MPx, myeloperoxidase; PAS, periodic acid-schiff, SBB; sudan black B; TB, toluidine blue). Basophils were not conclusively identified with the cytochemical stains, except for PAS.

Blood samples from eight wild caught clinically normal manatees (4 adults, 3 large calves, 1 subadult; 3 females, 5 males) were collected. Hematological data are reported in Table 1. The data was within ranges reported for Florida manatees, except for mildly increased nucleated red blood cells (nRBC) in one calf, which may be due to individual variation since calves tend to have higher numbers of nRBC compared to older life stages (7). WBC morphology was similar for all manatees across ages and sexes in the Wright-Giemsa-stained blood films, with exceptions noted below. A low number of bilobed mononuclear cells (1–3%, 0.04–0.36 K/μL) of undetermined origin was identified in Wright-Giemsa-stained blood films (Figure 1). White blood cells and platelets were further characterized by cytochemical staining (Figure 2 and Table 2).

**Table 1:** Hematological data from eight clinically normal, free-ranging Florida manatees (*Trichechus manatus latirostris*) obtained from EDTA-anticoagulated blood using an automated ADVIA 2120i hematology analyzer (Siemens Healthcare Diagnostics, Tarrytown, NY) and manual blood film review.

Tests		Subadult male	Adult male	Adult male	Adult male	Large calf male	Adult female	Large calf female	Large calf female
RBC	M/μL	2.73	3.27	3.34	2.73	3.21	2.91	2.83	3.69
HGB	g/dL	11.0	13.3	13.6	10.9	12.5	10.9	11.5	14.6
HCT	%	35	42	45	35	41	35	34	46
MCV	fL	127.6	127.8	133.5	128.0	127.6	118.6	120.3	123.8
MCH	pg	40.2	40.7	40.8	40.0	39.1	37.5	40.5	39.6
MCHC	g/dL	31.5	31.8	30.5	31.3	30.7	31.6	33.7	32.0
RDW	%	14.9	14.2	14.5	14.1	14.8	15.6	15.0	17.1
PCV (spun)	%	35	42	44	36	39	35	33	46
Plasma protein (refractometer)	g/dL	7.5	8.0	9.0	7.7	9.3	7.7	8.0	7.0
PLT	K/μL	222	372	501	283	543	210	210	367
MPV	fL	7.2	7.0	7.5	7.2	6.9	9.4	9.0	8.6
PLT estimate*		Adequate	Adequate	Adequate	Adequate	Adequate	Adequate	Adequate	Adequate
WBC	K/μL	4.66	4.39	7.23	3.65	12.01	6.46	5.04	6.46
WBC, corrected for nRBC	K/μL	4.66	4.39	7.23	3.65	12.01	6.46	5.04	5.93
Heterophils	%	36	51	54	51	48	35	48	46
	K/μL	1.70	2.30	3.90	1.90	5.80	2.30	2.40	2.70
Band heterophils	/μL	0	0	0	0	0	0	0	0
Eosinophils	%	2	5	4	2	2	3	6	3
	K/μL	0.09	0.22	0.29	0.07	0.24	0.20	0.30	0.18
Basophils	%	0	1	1	0	0	1	0	2
	K/μL	0.00	0.04	0.07	0.00	0.00	0.07	0.00	0.12
Lymphocytes	%	49	33	32	34	32	44	34	32
	K/μL	2.30	1.50	2.30	1.30	3.90	2.90	1.70	1.90
Monocytes	%	11	9	7	10	15	15	11	14
	K/μL	0.52	0.40	0.50	0.37	1.80	0.98	0.55	0.83
Bilobed monocytes	%	2	1	2	3	3	2	1	3
	K/μL	0.09	0.04	0.14	0.11	0.36	0.13	0.05	0.18
Nucleated RBC	/100 WBC	0	0	0	0	0	0	0	9
	/μL	0	0	0	0	0	0	0	533
WBC morphology		NSF	NSF	NSF	NSF	NSF	NSF	NSF	NSF
Heinz bodies (NMB stain)	%	2	1	3	2	0	7	9	16
RBC morphology		NSF	Rare schistocytes	NSF	NSF	Rare schistocytes and keratocytes	1+ anisocytosis	1+ anisocytosis, 1+ target cells	1+ anisocytosis, 1+ target cells, rare schistocytes

Blood films were evaluated via light microscopy with performance of a 200-leukocyte 421 differential count as per standard laboratory protocol.

* Small clumps were noted in all blood films.

HCT, hematocrit; HGB, hemoglobin; NSF, no significant findings; MCH, mean cell hemoglobin; MCHC, mean cell hemoglobin concentration; MCV, mean cell volume; MPV, mean platelet volume; NMB, new methylene blue; PCV, packed cell volume; PLT, platelet; RBC, red blood cell; RDW, red cell distribution width; WBC, white blood cell; EDTA, ethylenediaminetetraacetic acid.

**Table 2:** Cytochemical staining characteristics of leukocytes and platelets in Florida manatees (*Trichechus manatus latirostris*).

*n* = 8	ALP (*n* = 8)	ANBE (*n* = 8)	CAE (*n* = 8)	Luna (*n* = 3)	MPx (*n* = 8)	PAS (*n* = 3)	SBB (*n* = 8)	TB (*n* = 3)
Heterophils	G: +++	–	G: + to +++ C: - to + (diffuse)	–	G: + to ++	C: +++ (diffuse)	G: + to +++	–
Eosinophils	G: ++ to +++	–	–	G: +++	G: +++	C: ++ (diffuse)	+/−; G: + to ++	–
Lymphocytes	–	C: ++ to +++ (multifocal; most); − (few)	+/−; G: + to +++ (multifocal)	–	–	–	–	–
Monocytes	+/−; C: + (few punctate)	+/−; C: + to ++ (focal to diffuse)	+/−; C: + to ++ (focal to diffuse)	–	+/−; C: + to +++ (punctate)	+/−; C: + (diffuse)	+/−; C: + to +++ (punctate)	–
Bilobed monocytes	+/−; C: + (few punctate)	+/−; C: + (diffuse)	+/−; C: + (diffuse)	–	+/−; C: + (punctate)	+/−; C: + (diffuse)	+/−; C: + to ++ (punctate)	–
Platelets	–	C: +++ (punctate)	–	–	–	C: + to +++ (focal to multifocal)	–	–

Degree of staining was subjectively graded as positive (+), negative (−), equivocal (negative or weakly positive, +/−), weak (+), moderate (++) or strong (+++), in granules or cytoplasm, with the pattern of cytoplasmic staining further described as as punctate, focal, multifocal, or diffuse.

ALP, alkaline phosphatase; ANBE, alpha-naphthyl butyrate esterase; CAE, chloroacetate esterase; C, cytoplasm; G, granules; MPx, myeloperoxidase; PAS, periodic acid schiff; SBB, sudan black B; TB, toluidine blue. Erythrocytes were negative for each stain evaluated.

### Heterophils

Heterophils were the predominant WBC in manatees (mean 46%, range 36–54%). Heterophils measured ~12–16 μm in diameter and contained a segmented nucleus with 2–4 segments and clumped chromatin. They were characterized by abundant round, oval, or slightly elongated pink granules in a colorless to light blue cytoplasm in Wright-Giemsa-stained blood films. Cytochemical staining yielded strong ALP staining, variable (weak to strong) staining for CAE and SBB, and weak to moderate staining for MPx in the granules. The cytoplasm of heterophils was negative or weakly diffuse positive for CAE and strongly diffuse positive with slight granular appearance for PAS. Heterophils were negative for ANBE, Luna, and TB stains.

### Eosinophils

Eosinophils comprised a low percentage of the WBC population (mean 3%, range 2–6%). Eosinophils measured ~13 to 18 μm in diameter and contained a segmented nucleus with clumped chromatin. They contained round pink cytoplasmic granules in a colorless to pale blue cytoplasm. The granules were slightly larger and brighter pink than heterophil granules in Wright’s-Giemsa-stained blood films. Eosinophil granules had moderate to strong staining for ALP, strong positive staining for Luna and MPx and variable (negative to moderate positive) staining for SBB. They had moderate diffuse cytoplasmic positivity for PAS. MPx and PAS staining was subjectively stronger and weaker, respectively, in eosinophils compared to heterophils. Eosinophils were negative for ANBE, CAE, and TB stains.

### Basophils

Basophils were identified in very low numbers (mean 0.6%, range 0–2%). They measured ~13 to 14 μm in diameter and contained an indented nucleus with clumped chromatin. The cytoplasm was colorless and contained variable numbers of irregularly sized, round purple granules in Wright’s-Giemsa-stained blood films. Basophils were not convincingly identified with most of the cytochemical stains, except for PAS, where they appeared negative.

### Lymphocytes

Lymphocytes were the second most frequent WBC type following heterophils (mean 36%, 32–49%). Lymphocytes ranged from 9 to 13 μm in diameter (small to intermediate) with rare lymphocytes up to 15 to 16 μm (large lymphocytes). In Wright-Giemsa-stained blood films, the majority of lymphocytes were round to oval with a round to infrequently indented or lobulated nucleus, containing finely granular to clumped chromatin. They had a small rim of pale blue cytoplasm. Rare lymphocytes with red cytoplasmic granules were noted. On cytochemical staining, lymphocytes had moderate to strong chunky multifocal ANBE cytoplasmic staining. Lymphocytes stained negatively or showed multifocal weak positive granular staining in the cytoplasm with CAE. Lymphocytes were negative for ALP, Luna, MPx, PAS, SBB, and TB stains.

### “Classic” monocytes

Monocytes were noted in low to moderate numbers (mean 11.5%, range 7–15%). With the Wright-Giemsa stain, monocytes measured ~13 to 18 μm in diameter and had an irregular to reniform to oval nucleus with fine, lacy to slightly condensed chromatin. They had a moderate to abundant amount of pale blue to gray cytoplasm that infrequently contained a few small, punctate, non-staining vacuoles and/or occasional fine, pink granules. Cytochemical staining was variable, with individual cells being variably positive for all stains except negative staining for Luna and TB. Positive cytoplasmic staining reactions ranged from weak to moderate focal to diffuse positivity for ANBE and CAE, weak diffuse positivity for PAS, low numbers of weak positive granules for ALP, and weak to strong granular staining for MPx and SBB.

### Bilobed mononuclear cells

Bilobed mononuclear cells constituted about 1–3% (mean 2%) of the WBC differential count. With the Wright-Giemsa stain, bilobed mononuclear cells measured ~14–18 μm in diameter and contained indented to bilobed nuclei with lacy to condensed chromatin. They had a moderate amount of pale blue to gray cytoplasm that rarely contained a few small, punctate, non-staining vacuoles, similar to monocytes. Cytochemical staining resulted in negative to weakly positive staining for ALP, ANBE, CAE, MPx, PAS, and SBB. Cytochemical staining reactions largely mimicked those of monocytes, although reactions were judged weaker for most of the stains. Bilobed mononuclear cells were negative for Luna and TB. The staining characteristics of bilobed mononuclear cells support a monocyte origin for these cells.

### Platelets

Platelets were identified in small clumps or were scattered individually throughout the smears. They were round to oval with elongated forms and contained pink to red cytoplasmic granules in blood films stained with Wright-Giemsa. Platelets showed strong granular staining for ANBE and weak to strong granular focal to multifocal staining for PAS in their cytoplasm. They were negative for the other stains.

## Discussion

This study used morphological features in a routine hematological stain and a suite of cytochemical stains to characterize WBCs and platelets and classify WBC in manatees. Heterophils were the predominant WBC in manatees, which is in contrast to elephants in which monocytes predominate (12, 13). As in this study, heterophils and lymphocytes comprised the majority of leukocytes in previous studies evaluating free-ranging and captive manatees along with free-ranging dugongs (7, 26). Harvey et al. concluded that heterophils and lymphocytes are typically present in approximately equal numbers, with manatees in managed care (*n* = 62) having slightly but significantly higher absolute numbers of heterophils than free-ranging manatees (*n* = 52) (mean of 3.25 × 10^9^/L versus 2.33 × 10^9^/L, respectively) (7). The animals in this study were considered clinically normal based on physical examination and results of blood analyses. Comparing morphological features and cytochemical staining to other species in the same clade, manatee heterophils have similar granule staining compared to dugongs but smaller and less prominent granule staining compared to elephants and rock hyraxes in Wright’s-stained blood films (13). Cytochemical staining reactions in heterophils were more similar comparing manatees to elephants versus the dugong (Table 3).

**Table 3:** Cytochemical staining comparisons between manatees (*Trichechus manatus latirostris*), dugongs (*Dugong dugon*), and elephants.

	ALP	ANBE	CAE	Luna	MPx*	PAS	SBB	TB
Heterophils								
Manatee	+++	−	+ to +++	−	+ to ++	+++	+ to +++	−
Dugong^1^	−	+/− (−)	+/− (−)	NP	+	+	+/− (−)	NP
Elephant^2^^,^^3^	+	−	+	NP	+	+/−	++	NP
Eosinophils								
Manatee	++ to +++	−	−	+++	+++	++	+/− (+ to ++)	−
Dugong^1^	−	+	−	NP	+/−	+/−	+/−	NP
Elephant^2^^,^^3^	−	+	−	NP	+	−	−	NP
Lymphocytes								
Manatee	−	++ to +++	+/− (+ to +++)	−	−	−	−	−
Dugong^1^	+	+/−	−	NP	−	+/− (−)	−	NP
Elephant^2^^,^^3^	−	−	+/−	NP	−	−	−	NP
Monocytes								
Manatee	+/− (+)	+/− (+ to ++)	+/− (+ to ++)	−	+/− (+ to +++)	+/− (+)	+/− (+ to +++)	−
Dugong^1^	+/− (−)	+/−	−	NP	–	+/− (−)	−	NP
Elephant^2^^,^^3^	−	−	+/−	NP	+	NF	+	NP
Bilobed monocytes								
Manatee	+/− (+)	+/− (+)	+/− (+)	−	+/− (+)	+/− (+)	+/− (+ to ++)	−
Dugong^1^	NF	NF	NF	NF	NF	NF	NF	NF
Elephant^2^^,^^3^	−	−	+/−	NP	+	−	+	NP
Platelets								
Manatee	−	+++	−	−	−	+ to +++	−	−
Dugong^1^	NP	NP	NP	NP	NP	NP	NP	NP
Elephant^2^^,^^3^	NP	NP	NP	NP	NP	−	−	NP

Degree of staining was subjectively graded as positive (+), negative (−), equivocal (negative or weakly positive, +/−), weak (+), moderate (++) or strong (+++).

ALP, alkaline phosphatase; ANBE, alpha-naphthyl butyrate esterase; CAE, naphthol AS-D chloroacetate esterase; MPx, myeloperoxidase; PAS, periodic acid schiff; SBB, Sudan black B; TB, toluidine blue; NP, cytochemical stain not performed; NF, not found.

* MPx was evaluated in manatees and peroxidase (no details provided in referenced manuscripts) was evaluated in dugongs and elephants.

1 Woolford L, Wong A, Sneath HL, Long T, Boyd SP, Lanyon JM. Hematology of dugongs (*Dugong dugon*) in southern Queensland. Vet Clin Pathol. (2015) 44 (4):530–541.

2 Harr KE, Isaza R, Blue JT. Hematology of Elephants, In: Schalm’s Veterinary Hematology 6th edition. Eds: Douglass J Weiss and K. Jane Wardrop. Wiley-Blackwell, Ames, Iowa USA (2010) 942–949.

3 Salakij J, Salakij C, Narkkong N, et al. Hematology, cytochemistry and ultrastructure of blood cells from Asian elephant (*Elephas maximus*). Kasetsart J (Nat Sci). (2005) 39:482–493.

Eosinophils have previously been reported to stain positive for Luna, Luxol fast blue, MPx, and SBB with inconsistent staining with CAE or ANBE in other mammals (18, 24, 25, 27, 28). In this study, manatee eosinophils showed positive staining reactions for ALP, MPx, PAS, and SBB, which differs from that reported in elephants and dugongs (Table 3). They were also the only WBC that was positive with the Luna stain, indicating that this is a useful stain for differentiating eosinophils from heterophils in this species (Luna staining has not yet been evaluated in elephants or dugongs). Basophils were seen in too few numbers to ascertain their characteristic reactions with the cytochemical stains.

Lymphocytes in the manatees of this study were morphologically similar to other animals with a Wright-Giemsa stain, and absolute numbers were comparable to free ranging manatees evaluated in Harvey and colleagues and similar to that reported for elephants and dugongs (7, 12, 13, 26). Lymphocytes could be differentiated from monocytes in this study by negative staining reactions for ALP, MPx, PAS, and SBB, which is similar to elephant and dugong lymphocytes except that dugong lymphocytes stain positively for ALP and have variable positivity with PAS (Table 3).

In Wright-Giemsa-stained blood films, monocytes in the manatees of this study shared morphological characteristics with “classic” monocytes in other mammals although numbers are lower in manatees and dugongs than in elephants (7, 12, 13, 26). Monocyte cytochemical staining reactions largely differed in the manatee versus the elephant and dugong. In this study, monocytes were variably positive for all stains, except Luna and TB, whereas elephant monocytes are positive for MPx and variably positive for CAE and dugong monocytes can be positive for ANBE, ALP and PAS (12, 13, 26) (Table 3).

Bilobed monocytes have been identified in various species, particularly elephants, rock hyraxes, and giant pandas (12, 14, 26). Cytochemical staining for MPx and SBB has been previously used to aid in differentiating bilobed monocytes from lymphocytes in elephants and red pandas with monocytes being positive and lymphocytes being negative for these stains (12, 14) (Table 3). Cytochemical staining of the bilobed mononuclear cell identified in Wright-Giemsa-stained blood films of the manatees in this study yielded similar results to monocytes, indicating that bilobed mononuclear cells in manatees could be classified as a monocyte variant. It is suspected that these bilobed monocytes represent either a morphological variant with a bilobed nucleus that initially develops as a spherical nucleus and that subsequently acquires an indentation resulting in a separation of the lobes (29) or an acquired phenotypic variant (6). Manatees and rock hyraxes have far lower proportions of these cells (1–3%) compared to elephants, which can have between 20 and 60% bilobed monocytes (12, 13, 26). Bilobed monocytes were not described in a recent study of dugongs (26), again demonstrating species differences within the clade. We recommend these bilobed cells are counted as monocytes when performing a differential leukocyte count on Romanowsky-type stained blood films from manatees. Since their numbers are low in clinically normal free-ranging manatees, there are minimal consequences for not correctly identifying bilobed cells as monocytes in Wright-Giemsa-stained blood films. However, it is unknown if and how the proportions of these bilobed monocytes change with various diseases or clinical conditions in manatees. Monocytosis has been documented in manatees with cold-stress syndrome, although it is not known if the monocyte counts in that study included the bilobed variants (30). If the proportions of the latter cells increase with specific diseases, misclassification as lymphocytes may have more clinical relevance in manatees.

Manatee platelets had strong cytoplasmic granular positivity to ANBE and weak to strong positivity with PAS in this study. PAS positivity appears seen in platelets across many animal species, including thrombocytes of birds and reptiles, but is negative in elephants (13, 18, 31, 32) (Table 3). Nonspecific esterase staining has been previously identified in platelets of cats and dogs with positive staining of thrombocytes in green sea turtles and iguanas (19).

## Conclusion

In this study, we identified a type of leukocyte not reported in manatees to date, a bilobed mononuclear cell. These cells were classified as monocytes based on morphological and cytochemical staining characteristics, which is similar to other select species within the *Paenungula,* including Asian and African elephants and, based on morphological features alone, the rock hyrax. This new information will aid in determining accurate WBC differential cell counts in Florida manatees. Differences in cytochemical staining reactions for WBCs and platelets were seen in manatees of this study compared to elephants and dugongs. These differences can potentially be explained by species-specific expression of proteins or enzymes or the type of cytochemical stains used in each study. This study provides new information on the morphological features and cytochemical staining characteristics of Florida manatee WBCs and platelets. This information will aid in obtaining accurate hematological data for this species and is relevant for health assessment studies, diagnosis and monitoring of disease during rehabilitation, and clinical management of these animals.

## Data availability statement

The original contributions presented in the study are included in the article/supplementary material, further inquiries can be directed to the corresponding author.

## Ethics statement

This project was approved by the University of Florida’s Institutional Animal Care and Use Committee #202006823 and samples were collected under US Fish and Wildlife Service research permits #MA791721 and #MA067116-2.

## Author contributions

LC acquired the data, took the photomicrographs, compiled the image composites and tables, and drafted the manuscript. NS designed the study, acquired the samples, performed the Wright-Giemsa staining, analyzed the data, interpreted the cytochemical staining reactions, took photomicrographs, and wrote the manuscript. TS completed the cytochemical staining and interpretation, took photomicrographs, performed the data analysis, interpretation, and wrote the manuscript. JH assisted with the data interpretation and manuscript editing. MdW, LA, MW, and RB supported sample acquisition, provided logistical support, and edited the manuscript. All authors contributed to the article and approved the submitted version.

## Funding

This work was supported by a grant from the Florida Fish and Wildlife Conservation Commission.

## Conflict of interest

The authors declare that the research was conducted in the absence of any commercial or financial relationships that could be construed as a potential conflict of interest.

## Publisher’s note

All claims expressed in this article are solely those of the authors and do not necessarily represent those of their affiliated organizations, or those of the publisher, the editors and the reviewers. Any product that may be evaluated in this article, or claim that may be made by its manufacturer, is not guaranteed or endorsed by the publisher.

## Figures and Tables

**Table:** 

ALP	Alkaline phosphatase
ANBE	α-naphthyl butyrate esterase
CAE	Chloroacetate esterase
CBC	Complete blood count
EDTA	Ethylenediaminetetraacetic acid
HCT	Hematocrit
HGB	Hemoglobin
NSF	No significant findings
MCH	Mean cell hemoglobin
MCHC	Mean cell hemoglobin concentration
MCV	Mean cell volume
MPV	Mean platelet volume
MPx	Myeloperoxidase
NMB	New methylene blue
nRBC	Nucleated red blood cell
PAS	Periodic acid-Schiff
PCV	Packed cell volume
PLT	Platelet
RBC	Red blood cell
RDW	Red cell distribution width
SBB	Sudan black B
TB	Toluidine blue
WBC	White blood cell
